# Use of Approximate Bayesian Computation to Assess and Fit Models of *Mycobacterium leprae* to Predict Outcomes of the Brazilian Control Program

**DOI:** 10.1371/journal.pone.0129535

**Published:** 2015-06-24

**Authors:** Rebecca Lee Smith, Yrjö Tapio Gröhn

**Affiliations:** 1 Department of Pathobiology, University of Illinois College of Veterinary Medicine, Urbana, Illinois, United States of America; 2 Department of Population Medicine and Diagnostic Science, Cornell University College of Veterinary Medicine, Ithaca, New York, United States of America; National Taiwan University, TAIWAN

## Abstract

Hansen’s disease (leprosy) elimination has proven difficult in several countries, including Brazil, and there is a need for a mathematical model that can predict control program efficacy. This study applied the Approximate Bayesian Computation algorithm to fit 6 different proposed models to each of the 5 regions of Brazil, then fitted hierarchical models based on the best-fit regional models to the entire country. The best model proposed for most regions was a simple model. Posterior checks found that the model results were more similar to the observed incidence after fitting than before, and that parameters varied slightly by region. Current control programs were predicted to require additional measures to eliminate Hansen’s Disease as a public health problem in Brazil.

## Introduction

Hansen’s disease (HD, or leprosy) is caused by chronic infection with *M*. *leprae*. After initial infection, most likely through respiratory droplet spread or direct contact [1], infected individuals enter a latent period of varying length, generally thought to be 3–5 years [1]. Upon becoming symptomatic, individuals will become either paucibacillary (PB, having 5 or fewer skin lesions) or multibacillary (MB, more than 5 skin lesions), possibly due to genetic factors in host immune response [1]. The 2 forms of the disease result in differing levels of peripheral nerve damage caused by the immune response to infection, with MB individuals suffering from higher grades of disability and more social stigma [2]. In contrast, PB individuals may heal spontaneously [1]. Diagnosis and classification are generally based on clinical signs, as diagnostic tests are limited.

The World Health Organization has set a goal of eliminating HD as a public health problem, defined as an annual incidence rate of <1/10,000 [3]. Despite global success at reaching this goal, clusters of high prevalence remain in at least 9 countries [4], including Brazil [5–7]. In these countries, models may assist in identifying the most effective control points. Generalized mathematical modeling of HD has been limited to a series of discrete-time Reed-Frost models [8,9] and deterministic compartmental models that have not been fitted to data [10–12]. An individual-based model has been produced [9], but is specific to the demographic situation in Bangladesh. Model predictions of the contribution of any control program to changes in incidence has been found to be highly dependent on the assumptions of the model, many of which are the result of persistent gaps in knowledge [13]. There is a need for a model that will predict the effects of control strategies accurately [14], as the role of interventions such as vaccines is debated [15]. In particular, the transmission rate is difficult to determine, as screening tests are not available and case detection is known to vary by region and over time [16]. The rate of transition from latency to symptomatic disease, as well, is difficult to determine, as direct estimation would rely on knowing the time of infection, which is especially unlikely in household or community acquired infections.

Approximate Bayesian Computation (ABC) has been applied to several infectious disease models [17–20] with great success, as the likelihood-free approach allows for application to complicated models where the explicit likelihood function is intractable [21]. The ABC algorithm also allows for direct comparison of different models. The purpose of this study is to use the ABC algorithm to better fit existing models of HD to incidence data from Brazil and to select the model with the best fit to the observed data. This model can then be used to predict the effect of control strategies.

## Materials and Methods

### Existing Models and Assumptions

**Fig 1:**
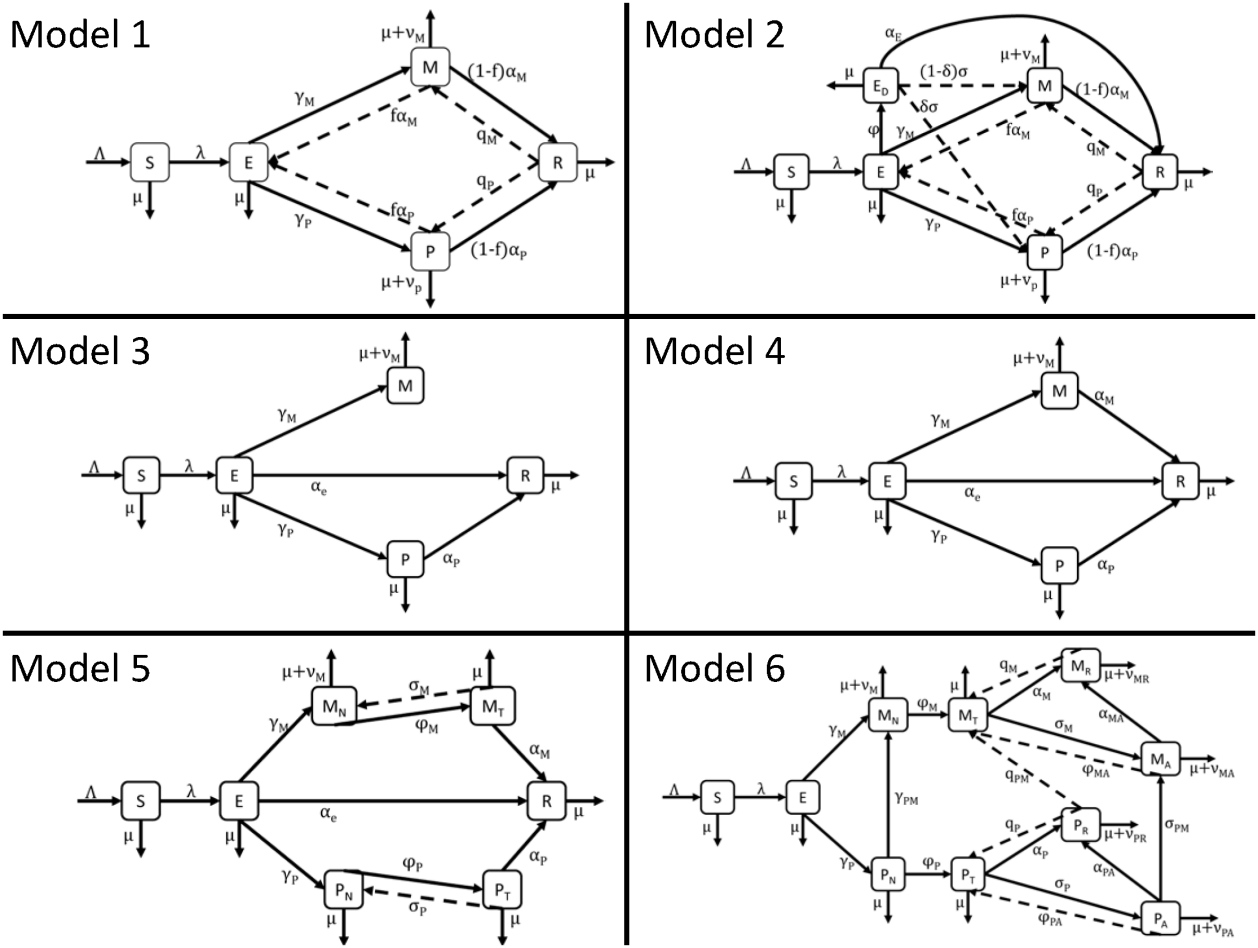
A schematic of the compartment models for *M*. *leprae*.

Deterministic compartment models for *M*. *leprae* (Fig 1) were previously developed and analyzed mathematically [8,10–12]. Regional model parameters are listed in Table 1 and model-specific parameters are listed in Table 2; all parameters are based on the parameters used by the developers of the models. State names (i.e., S or E) are used to refer to the number of individuals in that state.

**Table 1:** Regional parameters used to model Hansen’s Disease in Brazil.

Region	Population size^5^ in 2000	Annual Growth Rate^5^ (/10,000) in 2000–2010 (*Λ-μ*)	Annual Death Rate^5^ (/10,000) in 2000–2010 (*μ*)	Prevalence of Hansen’s Disease^5^ (/10,000) in 2000
North	13,223,859	0.021	0.50	8.73
Northeast	48,332,163	0.011	0.66	6.92
Southeast	73,501,405	0.011	0.64	2.87
South	24,442,941	0.0087	0.62	1.35
Midwest	11,881,087	0.019	0.49	9.83

**Table 2:** Starting parameter values for 6 models of Hansen’s Disease.

Symbol		Value					
	Description Model	1	2	3	4	5	6
Λ	rate at which susceptibles enter the population	regional (growth rate + death rate)					
μ	mortality rate	regional (death rate)					
β_P_	effective contact rate for PB	0.15 (0–0.95)					
β_M_	effective contact rate for MB	0.3 (0–0.95)					
θ_1_	reduction factor of β for treated over untreated PB					0.74	0.02
θ_2_	reduction factor of β for treated over untreated MB					0.74	0.02
θ_3_	reduction factor of β for recalcitrant over untreated MB						0.18
γ_M_	rate of progression to MB	0.1 (0–0.4)					
γ_P_	rate of progression to PB	0.2 (0–0.4)					
γ_PM_	rate of progression from untreated PB to MB						0.0017
δ_E_	fraction of progressing individuals becoming PB		0.5				
α_M_	recovery rate from MB	0.2	0.2		0.2	0.2	0.2
α_MA_	recovery rate from recalcitrant MB						0.1
α_P_	recovery rate from PB	0.3					
α_PA_	recovery rate from recalcitrant PB						0.17
α_E_	recovery rate from latent		0.65	0.56	0.56	0.56	
q_M_	relapse rate to MB	0.06	0.06				0.02
q_P_	relapse rate to PB	0.1	0.1				0.01
q_PM_	relapse rate from PB to MB						0.0012
φ_E_	case finding rate for latent		0.5				
φ_M_	case finding rate for MB					0.02	0.5
φ_MA_	case finding rate for recalcitrant MB						0.32
φ_P_	case finding rate for PB					0.04	0.5
φ_PA_	case finding rate for recalcitrant PB						0.14
f	fraction of individuals who fail to complete active treatment	0.1	0.1				
σ_E_	rate of progression from latent despite treatment		0.1				
σ_M_	rate of relapse from MB despite treatment					0.1	0.1
σ_P_	rate of relapse from PB despite treatment					0.1	0.1
σ_PM_	rate of progression from PB to MB despite treatment						0.002
v_M_	disease-induced mortality rate in MB	0.05					
ν_MA_	disease-induced mortality rate in recalcitrant MB						0.04
ν_MR_	disease-induced mortality rate in recovered MB						0.01
v_P_	disease-induced mortality rate in PB	0.2	0.2				0.009
ν_PA_	disease-induced mortality rate in recalcitrant PB						0.014
ν_PR_	disease-induced mortality rate in recovered PB						0.009

PB: paucibacillary

MB: multibacillary

All models assume that individuals enter the population as susceptible (S) and, after infection at rate λ, become latently infected (E). In Model 1 [10], latent individuals may spontaneously enter the recovered state (R), or they may become clinically diseased in the multibacillary state (M), where they are subject to an extra mortality rate ν_M_, or paucibacillary (P) state. Model 1 assumes that all infected individuals can recover and will not relapse. In Model 2 [11], latent individuals may become detected (E_D_), at which point they may recover (R) due to treatment. Both undetected and detected individuals may become multibacillary (M) or paucibacillary (P), from which they may recover or return to undetected latency. Model 2 assumes that recovered individuals (R) may relapse to either multibacillary or paucibacillary disease. Model 3 [12] is identical to Model 1 except for the assumption that multibacillary individuals (M) do not recover. Model 4 [12] is identical to Model 2 except for the assumption that latent cases (E) cannot be detected and cannot recover. The transmission rate, λ, is calculated in Models 1, 2, 3, and 4 as $\lambda =\frac{\beta_{P}P+\beta_{M}M}{N}$, where *β*_*P*_ is the transmission coefficient for paucibacillary individuals and *β*_*M*_ is the transmission coefficient for multibacillary individuals. Model 5 [12] divides the multibacillary and paucibacillary compartments into untreated (M_N_, P_N_) and treated (M_T_, P_T_) states, with the assumption that treated multibacillary cases do not suffer from extra mortality. Model 5 [12] assumes that treated individuals may recover (R) or relapse to the untreated state. The transmission rate, λ, in Model 5 as $\lambda =\frac{\beta_{P}\left(P_{N}+\theta_{1}P_{T}\right)+\beta_{M}\left(M_{N}+\theta_{2}M_{T}\right)}{N}$, where θ_i_ is the proportional decrease in infectiousness associated with treatment of disease type i. Model 6 [8] also divides the diseased compartments into untreated and treated states, and assumes that untreated paucibacillary disease may develop into multibacillary disease. Model 6 also assumes: treated individuals may enter a recovered category (M_R_, P_R_) or a dormant disease category (M_A_, P_A_), with either category able to relapse to the treated category; dormant paucibacillary disease (P_A_) may develop into dormant multibacillary disease (M_A_), and that recovered paucibacillary disease (P_R_) may relapse into treated multibacillary disease (M_T_); and several disease categories are subject to an extra mortality rate, ν_i_ for each category i, *i*∈{*M*_*N*_,*M*_*R*_,*M*_*A*_,*P*_*R*_,*P*_*A*_} The transmission rate, λ, in Model 6 as $\lambda =\frac{\beta_{P}\left(P_{N}+\theta_{1}P_{T}+\theta_{3}P_{A}\right)+\beta_{M}\left(M_{N}+\theta_{2}M_{T}+\theta_{3}M_{A}\right)}{N}$. The birth rate, Λ, was assumed to be the reported growth rate plus the death rate (Table 1).

The initial number of individuals in each category was determined empirically (see S1 File) in relation to the initial prevalence and the assumed proportion of MB disease in existing cases, *P(MB)*. To calculate *P(MB)*, the average proportion of MB cases among existing cases was calculated across each region for all years. The calculations used in each model are shown in Table 3.

**Table 3:** Assumptions made about the initial number of individuals per category and the calculation of incidence for 6 models of Hansen’s Disease.

		Model					
Category	Description	1	2	3	4	5	6
S	susceptible	N-(E+M+P+R)	N-(E+E_D_+M+P+R)	N-(E+ M+P+R)		N-(E+M+M_2_+P+ P_2_+R)	N-(E+M+M_A_+M_R_+P+ P_A_+P_R_)
E	latent	π_e_*C					
E_D_	detected latent		π_ed_*C				
M/M_N_	MB (all or untreated)	C*P(MB)*P_m_				π_e_*C*P(MB)	π_e_*C*P(MB)
M_T_	treated MB					C*P(MB)	π_mt_*C*P(MB)
M_A_	recalcitrant MB						(1-π_mt_)*C*P(MB)
M_R_	recovered MB						π_R,M_*M_T_
P/P_N_	PB (all or untreated)	C-M				π_e_*C	π_e_*C
P_T_	treated PB					C*(1-P(MB))	π_pt_*(C-M_T_-M_A_)
P_A_	recalcitrant PB						(1-π_pt_)*(C-M_T_-M_A_)
P_R_	recovered PB						π_R,P_*P_T_
R	recovered	π_R_*C	π_R_*C	π_R_*C	π_R_*C	π_R_*C	
PB Incidence		γ_p_E+q_p_R	σδE_D_+γ_p_E+q_p_R	γ_p_E		q_p_P_N_	φ_p_P_N_+q_P_P_R_+φ_PA_P_A_
MB Incidence		γ_M_E+q_M_R	σ(1-δ)E_D_+γ_M_E+q_M_R	γ_M_E		q_M_M_N_	σ_M_M_N_+q_PM_P_R_+φ_MA_M_A_+q_M_M_R_
Apparent Prevalence		(M + P)/N	(M + P + E_D_)/N	(M + P)/N		(M_T_ + P_T_)/N	(M_T_ + M_A_ + P_T_ + P_A_)/N

PB: paucibacillary

MB: multibacillary

N: regional population size

C: number of cases expected (N*prev)

prev: reported regional prevalence in 2000

P(MB): observed regional probability that a new case is multibacillary

π_i_: relationship between C and the initial number of individuals in the *i* compartment (see S1 File)

### Approximate Bayesian Computation

The ABC algorithm used to fit each model was identical, following the Sequential Monte Carlo algorithm proposed by Toni et al. [22] The distance function was calculated as

$$d=\sum_{t=2000}^{2012}{\left(inc^{PB}\left(t\right)-inc_{fit}^{PB}\left(t\right)\right)}^{2}+{\left(inc^{MB}\left(t\right)-inc_{fit}^{MB}\left(t\right)\right)}^{2}$$ (1)

where *inc* ^*PB*^ *(t)* is the observed incidence of paucibacillary HD at time t, $inc_{fit}^{PB}\left(t\right)$ is the fitted incidence of paucibacillary HD at time t, *inc* ^*MB*^(*t*) is the observed incidence of multibacillary HD at time t, and $inc_{fit}^{MB}\left(t\right)$ is the fitted incidence of multibacillary HD at time t. The model-specific calculations for $inc_{fit}^{PB}\left(t\right)$ and $inc_{fit}^{MB}\left(t\right)$ are shown in Table 3. The first set was run without a rejection step, in order to empirically determine the tolerance. In each following set, the tolerance was set to the 60^th^ quantile of the distance function from the previous set [23] and the perturbation kernel for each parameter was set equal to a uniform distribution with a range of twice the variance of that parameter’s values from the previous set. Unknown parameters were the transmission parameters (β_M_ and β_P_) and transition parameters (γ_M_ and γ_P_) for MB and PB cases, respectively. Uniform prior distributions for the unknown parameters were based on a range that included the biological minimum (0) and at least twice the maximum value used by the developers of the models being fitted (Table 2). The algorithm was implemented for 10 sets of 100,000 iterations each. Validation of the model is presented in **S2**.

### Model Selection and Parameterization

**Fig 2:**
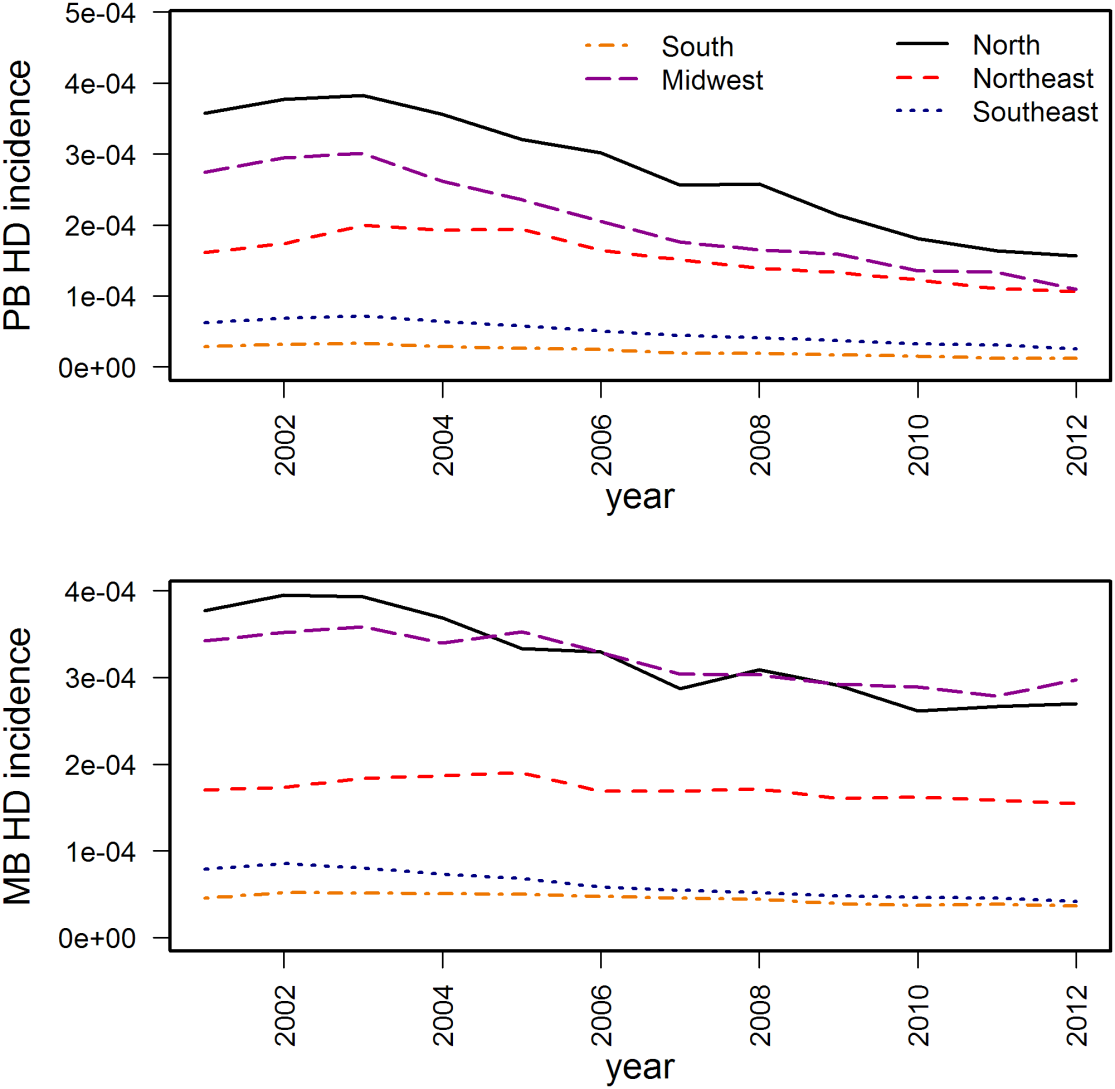
Annual incidence of Hansen’s Disease (HD) diagnosis in Brazil, by region [6]. The top graph is the total number of new cases of paucibacillary (PB) disease, while the bottom graph is the number of new cases of multibacillary (MB) disease.

**Fig 3:**
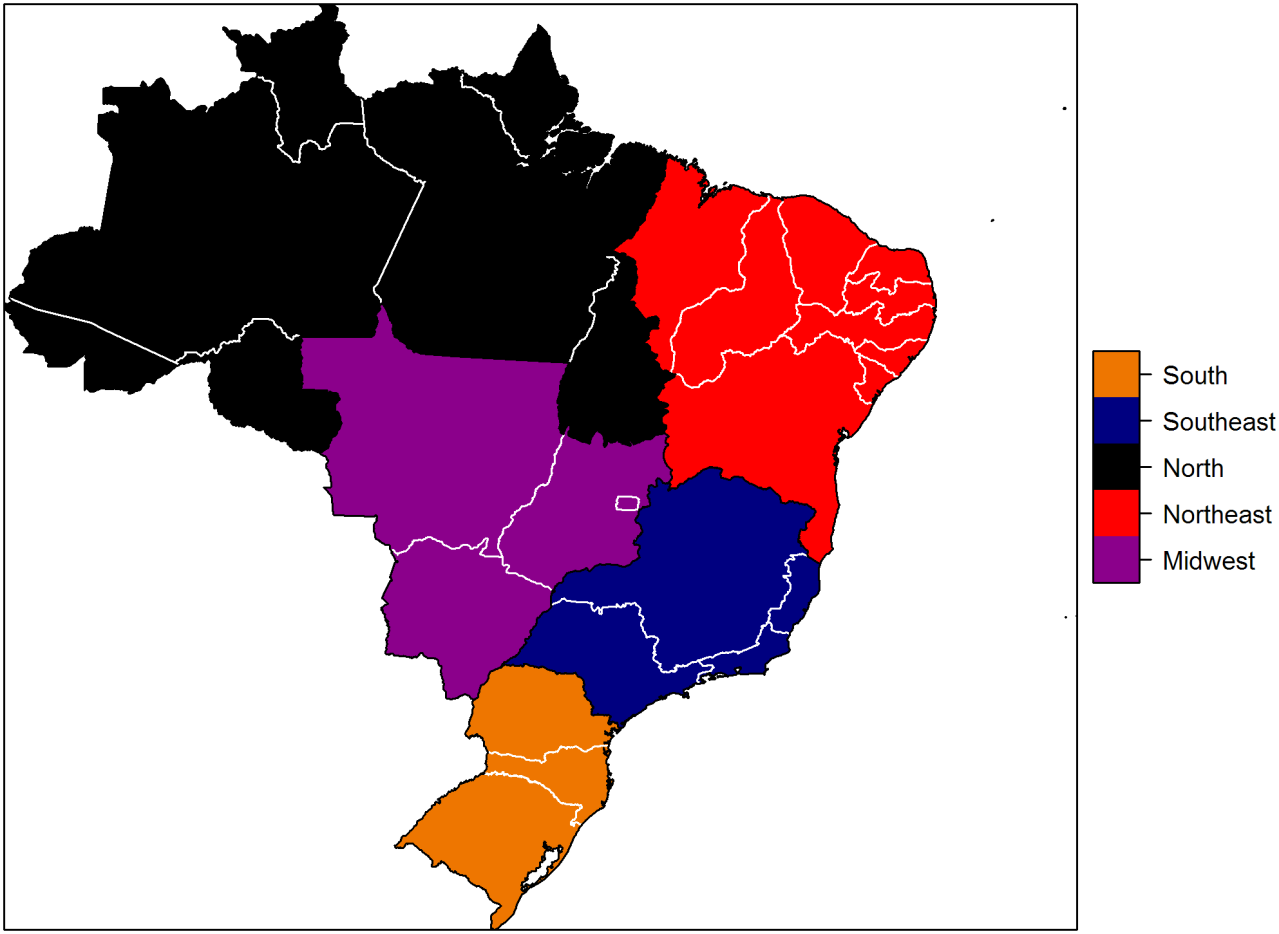
Posterior distribution of parameters for the best model of Hansen’s Disease by region of Brazil. All results are from Model 3. The top right graph shows the transmission parameter for multibacillary cases, the top left graph shows the transmission parameter for paucibacillary cases, the bottom left graph shows the progression rate for multibacillary cases, and the bottom right graph shows the progression rate for paucibacillary cases. Each line represents a different region: North (black solid), Northeast (NE, red dashed), South (blue dots), Southeast (SE, orange dot-dash), and Midwest (MW, purple long dash).

The models were fit and model selection was performed using incidence data from Brazil, which has endemic HD in all regions, from 2000 to 2012 [6]. These data include the population size, growth and death rates (Table 1), and the annual number of new PB and MB cases by region for the period of 2000 to 2012 (Table 4). Over this period, the number of cases declined in all regions (Fig 2). The regions represented in these data are shown in Fig 3.

**Table 4:** Regional incidence observations (cases per 10,000) used to fit models of Hansen’s Disease in Brazil.

	North		Northeast		Southeast		South		Midwest	
Year	PB	MB	PB	MB	PB	MB	PB	MB	PB	MB
2001	3.60	3.80	1.60	1.70	0.62	0.79	0.29	0.45	2.70	3.40
2002	3.80	4.00	1.70	1.70	0.68	0.85	0.32	0.52	2.90	3.50
2003	3.80	3.90	2.00	1.80	0.71	0.80	0.33	0.51	3.00	3.60
2004	3.60	3.70	1.90	1.90	0.64	0.73	0.28	0.51	2.60	3.40
2005	3.20	3.30	1.90	1.90	0.57	0.68	0.26	0.50	2.40	3.50
2006	3.00	3.30	1.60	1.70	0.50	0.59	0.25	0.47	2.10	3.30
2007	2.60	2.90	1.50	1.70	0.44	0.55	0.19	0.45	1.80	3.00
2008	2.60	3.10	1.40	1.70	0.41	0.52	0.19	0.45	1.70	3.00
2009	2.10	2.90	1.30	1.60	0.37	0.48	0.17	0.39	1.60	2.90
2010	1.80	2.60	1.20	1.60	0.32	0.46	0.15	0.38	1.40	2.90
2011	1.60	2.70	1.10	1.60	0.31	0.45	0.12	0.39	1.30	2.80
2012	1.60	2.70	1.10	1.60	0.25	0.42	0.12	0.37	1.10	3.00

PB: paucibacillary

MB: multibacillary

We used the ABC algorithm described above to estimate the parameters for each region and each model. Model selection was performed for each region as described above, with a Bayes Factor >1 for each model comparison indicating the preferred model. Distance values from the final set were summed for each model fitted, and each Bayes Factor was calculated as the ratio of the summed distance of each model to the summed distance of the comparative model. This provides the pairwise strength of evidence to prefer a particular model to the comparative model. Posterior distributions were determined for each of the 6 models in each of the 5 regions.

### Hierarchical Model Selection and Parameterization

After the model selection and parameterization process selected the best-fit model for each region, several hierarchical models were considered to allow for parameter distributions to be fit across regions. In version 1, all 4 parameters were shared across models and regions. In version 2, only transmission parameters were shared across regions. In version 3, only transition parameters were shared across regions. Version 4 consisted of the regionally-fit model. The ODE model used for hierarchical models was the model preferred by the majority of the regions. As different regions contained different numbers of infected individuals, the distance function was standardized by observed incidence to evenly weight the regions in fitting:

$$d*=\sum_{r}\left[\sum_{t=2000}^{2012}\frac{{\left(inc_{r}^{PB}\left(t\right)-inc_{r,fit}^{PB}\left(t\right)\right)}^{2}}{inc_{r}^{PB}\left(t\right)}+\frac{{\left(inc_{r}^{MB}\left(t\right)-inc_{r,fit}^{MB}\left(t\right)\right)}^{2}}{inc_{r}^{MB}\left(t\right)}\right]$$ (2)

where *r* represents the region. The ABC algorithm was implemented with sets of 10,000 iterations, but the number of sets was varied by the number of free parameters, 5 sets per parameter estimated. The priors used were the same as for the regional model selection and parameterization. The posterior distributions of each model (3 hierarchical models and the full regional model) were used to fit 100 iterations for each region. Model fit was determined by choosing the hierarchical model with the smallest summed d* over all regions in the posterior sample fits. For each region, a random weighted selection of 1,000 iterations from the best-fit model’s posterior distributions was used to calculate the effective reproduction rate (R_e_), the predicted prevalence in the region ihn 2050 (p_2050_), and the time necessary to eliminate HD from the region (t_elim_), which was calculated by solving the ODE model over 100 years and finding the earliest time at which the apparent prevalence fell below the WHO threshold of 0.0001, or 1 in 10,000[24].

In order to check the consistency of the model results, data were simulated for each region using the median of the best fitted value from the hierarchical model, using Model 3. These data were then used to repeat the full model selection and parameterization process, including hierarchical model selection and parameterization. Results were compared to the simulated input values.

## Results

The validation trials presented in the supplement found that the ABC algorithm used was able to reproduce many of the simulated values if moderate values were simulated, especially if Model 3 or Model 4 was used to simulate the data, but tended to produce posterior distributions closer to the center of the prior distribution if extreme values were simulated. The model selection process was able to identify the simulated model in most cases.

The Bayes Factor ratios from applying the ABC algorithm with each model to individual regions of Brazil are shown in Table 5, with the columns being the comparative models. For all but the Southern region, Model 3 provided the best fit, and no model was strongly preferred over Model 3 in the Southern region (although Model 4 was weakly preferred); therefore, all hierarchical modeling was performed on Model 3.

**Table 5:** Bayes Factor ratios comparing each model of Hansen’s Disease, by region of Brazil, as fitted by Approximate Bayesian Computation.

	Fitted	Comparative Model					
Region	Model	Model 1	Model 2	Model 3	Model 4	Model 5	Model 6
North	Model 1	*1*.*00*	*4*.*75*	0.05	0.24	0.71	*7*.*72*
	Model 2	0.21	*1*.*00*	0.01	0.05	0.15	*1*.*62*
	Model 3	*20*.*59*	**97.88**	*1*.*00*	*4*.*98*	*14*.*70*	**158.97**
	Model 4	*4*.*14*	*19*.*66*	0.20	*1*.*00*	*2*.*95*	**31.93**
	Model 5	*1*.*40*	*6*.*66*	0.07	0.34	*1*.*00*	*10*.*81*
	Model 6	0.13	*0*.*62*	0.01	0.03	0.09	*1*.*00*
Northeast	Model 1	*1*.*00*	*4*.*10*	0.05	0.46	0.48	0.38
	Model 2	0.24	*1*.*00*	0.01	0.11	0.12	0.09
	Model 3	*20*.*17*	**82.65**	*1*.*00*	*9*.*36*	*9*.*67*	*7*.*76*
	Model 4	*2*.*16*	*8*.*83*	0.11	*1*.*00*	*1*.*03*	0.83
	Model 5	*2*.*09*	*8*.*54*	0.10	0.97	*1*.*00*	0.80
	Model 6	*2*.*60*	*10*.*65*	0.13	*1*.*21*	*1*.*25*	*1*.*00*
Southeast	Model 1	*1*.*00*	*13*.*44*	0.36	*2*.*65*	*2*.*45*	*4*.*39*
	Model 2	0.07	*1*.*00*	0.03	0.20	0.18	0.33
	Model 3	*2*.*78*	**37.32**	*1*.*00*	*7*.*36*	*6*.*81*	*12*.*20*
	Model 4	0.38	*5*.*07*	0.14	*1*.*00*	0.93	*1*.*66*
	Model 5	0.41	*5*.*48*	0.15	*1*.*08*	*1*.*00*	*1*.*79*
	Model 6	0.23	*3*.*06*	0.08	0.60	0.56	*1*.*00*
South	Model 1	*1*.*00*	*7*.*02*	*1*.*78*	0.30	0.84	*1*.*88*
	Model 2	0.14	*1*.*00*	0.25	0.04	0.12	0.27
	Model 3	0.56	*3*.*94*	*1*.*00*	0.17	0.47	*1*.*05*
	Model 4	*3*.*37*	*23*.*64*	*6*.*00*	*1*.*00*	*2*.*82*	*6*.*32*
	Model 5	*1*.*19*	*8*.*39*	*2*.*13*	0.35	*1*.*00*	*2*.*24*
	Model 6	0.53	*3*.*74*	0.95	0.16	0.45	*1*.*00*
Midwest	Model 1	*1*.*00*	*2*.*68*	0.01	0.07	0.22	*3*.*60*
	Model 2	0.37	*1*.*00*	0.01	0.03	0.08	*1*.*34*
	Model 3	**73.01**	**195.78**	*1*.*00*	*5*.*27*	*16*.*42*	**263.09**
	Model 4	*13*.*86*	**37.17**	0.19	*1*.*00*	*3*.*12*	**49.95**
	Model 5	*4*.*45*	*11*.*92*	0.06	0.32	*1*.*00*	*16*.*02*
	Model 6	0.28	0.74	0.00	0.02	0.06	1.00

Each value is a pairwise comparison of the strength of evidence for the fitted model (row) against a comparative model (column). Values in bold were considered strongly in favor of the model represented in that row over the comparative model, while values in italics are considered weak.

**Fig 4:**
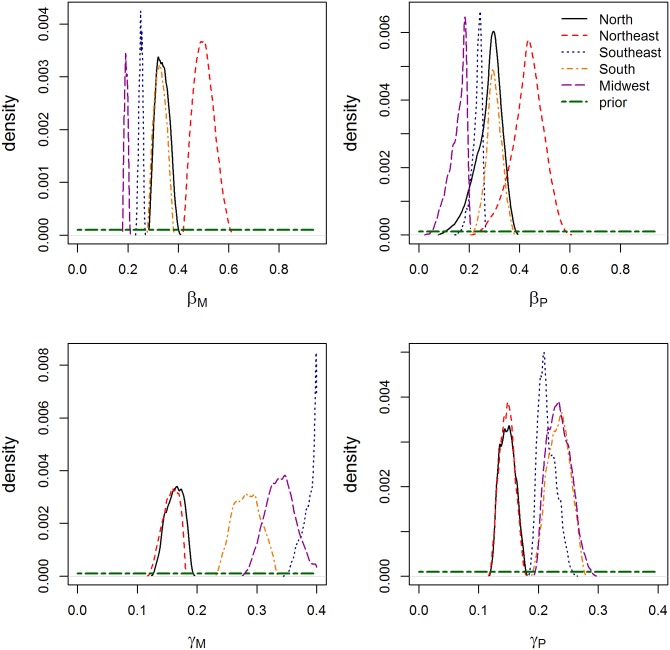
Map of Brazil, showing administrative regions.

The posterior distributions of the best fit models for each region are shown in Fig 4, compared to the prior distribution, and Table 6. The fitted transmission rate for PB cases, β_P_, is similar across regions, with overlapping posterior distributions. The fitted transmission rate for MB cases, β_M_, is more varied, with higher fitted values in the Northeast and lower fitted values in the Midwest and Southeast. Transition rates (γ_M_ and γ_P_), in contrast, show 2 distinct groupings of regionally fit parameters. The posterior distributions of the North and Northeast are significantly lower than that of other regions, especially for MB cases.

**Table 6:** Posterior distribution median and 95% prediction intervals determined by ABC fitting of Approximate Bayesian Computation models for Hansen’s Disease to data from the 5 regions of Brazil.

Version^a^	Region	β_M_	β_P_	γ_M_	γ_P_	Relative weight
1	All	0.24 (0.16–0.40)	0.17 (0–0.39)	0.29 (0.14–0.40)	0.19 (0.08–0.35)	8.6
	North			0.23 (0–0.40)	0.21 (0–0.40)	
	Northeast			0.27 (0–0.40)	0.25 (0–0.40)	
2	Southeast	0.23 (0.14–0.54)	0.18 (0–0.44)	0.28 (0–0.40)	0.25 (0–0.40)	1.3
	South			0.27 (0–0.40)	0.18 (0–0.40)	
	Midwest			0.23 (0–0.40)	0.15 (0–0.40)	
	North	0.43 (0–0.95)	0.2 (0–0.95)			
	Northeast	0.58 (0–0.95)	0.26 (0–0.95)			
3	Southeast	0.56 (0–0.95)	0.26 (0–0.95)	0.11 (0.03–0.40)	0.08 (0.02–0.40)	1
	South	0.39 (0–0.95)	0.18 (0–0.95)			
	Midwest	0.35 (0–0.95)	0.17 (0–0.87)			
	North	0.34 (0.28–0.41)	0.29 (0.08–0.39)	0.16 (0.12–0.20)	0.15 (0.12–0.18)	
	Northeast	0.5 (0.42–0.62)	0.44 (0.21–0.61)	0.16 (0.12–0.18)	0.15 (0.12–0.18)	
4	Southeast	0.25 (0.23–0.27)	0.24 (0.14–0.27)	0.39 (0.34–0.40)	0.21 (0.18–0.27)	5.2
	South	0.33 (0.28–0.38)	0.3 (0.20–0.38)	0.28 (0.23–0.33)	0.23 (0.19–0.28)	
	Midwest	0.19 (0.18–0.21)	0.16 (0.02–0.21)	0.34 (0.28–0.40)	0.23 (0.19–0.30)	

Version 4 consisted of fitting the regional best-fit model to each region’s observed data separately; all other versions used a hierarchical structure in which at least some parameters were shared across regions, and fitting was done simultaneously across all 5 regions. Relative weight refers to the Bayes Factor of each version when compared to the version with the worst fit (Version 3).

^a^Version of the hierarchical structure sharing parameters across 5 regions of Brazil: 1) all parameters shared; 2) transmission parameters shared; 3) transition parameters shared; 4) no parameters shared

The posterior distributions of the hierarchical models are shown in Table 6. As shown by the relative weight value in Table 6, Version 1, in which all parameters were shared across regions, was preferred to all other versions, with the regional model being the second-best fit. As would be expected, the hierarchical model posterior distributions fell in the midst of the regional posterior distributions, with no evidence that any one region was overly influential. Versions 2 and 3, in which some parameters were shared across regions, proved to have difficulty in fitting the regional parameters; posterior distributions were similar to prior distributions.

**Fig 5:**
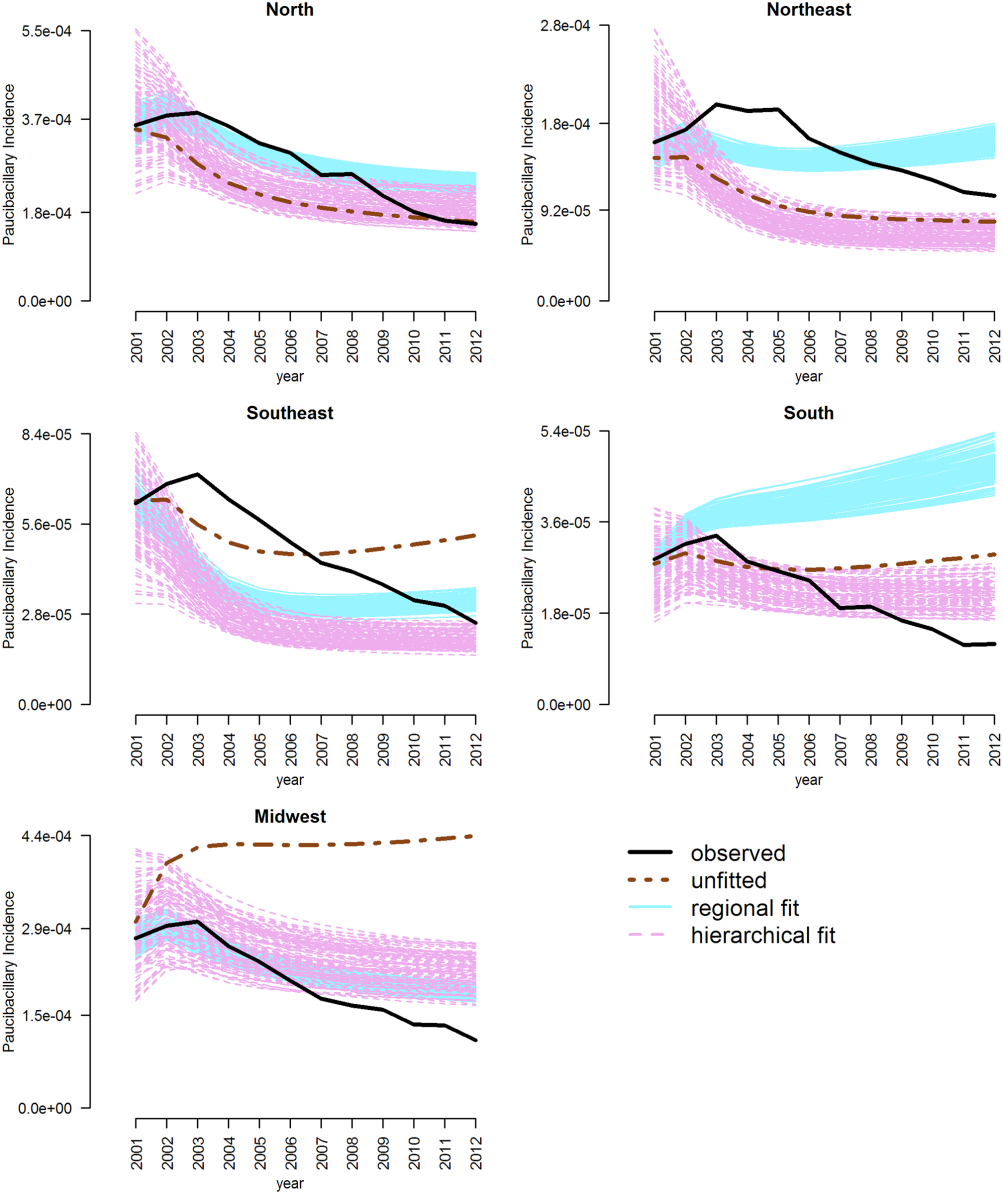
Posterior checks for the incidence of paucibacillary Hansen’s Disease in the 5 regions of Brazil. Observed incidence (black solid line) is shown with estimated incidence from Model 3 fitted hierarchically (purple dashed lines) and regionally (blue solid lines) and unfitted (brown dot-dash line). In the hierarchical model, parameters were fitted across all regions.

**Fig 6:**
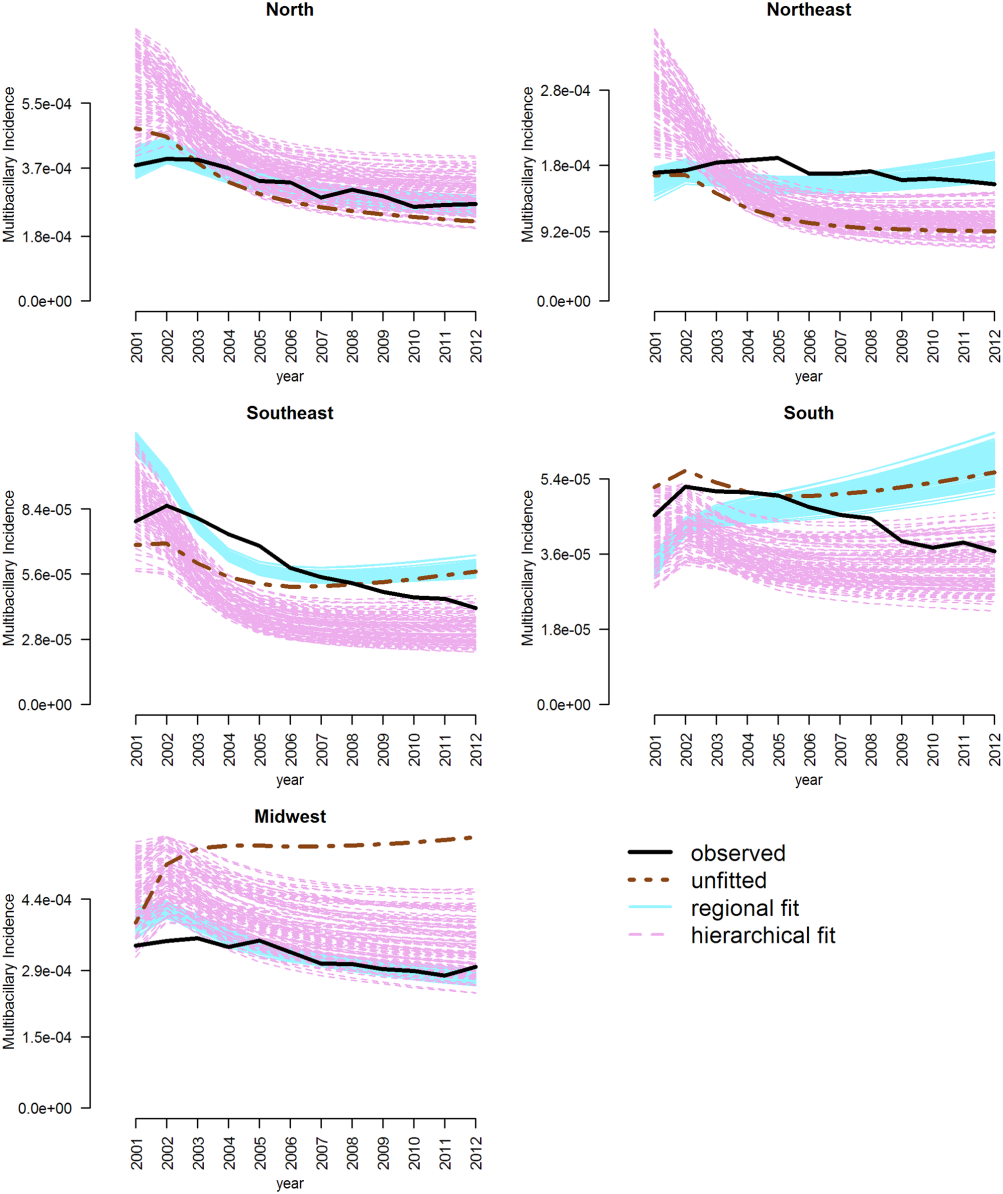
Posterior checks for the incidence of multibacillary Hansen’s Disease in the 5 regions of Brazil. Observed incidence (black solid line) is shown with estimated incidence from Model 3 fitted hierarchically (purple dashed lines) and regionally (blue solid lines) and unfitted (brown dot-dash line). In the hierarchical model, parameters were fitted across all regions.

The posterior estimations for incidence of PB and MB HD in all regions of Brazil, for both the best-fit regional model and the best-fit hierarchical model, are compared in Figs 5 and 6 to the incidence estimations of the unfitted model (using the parameters provided by the developers of the model) and the observed values. In most regions, the hierarchical model captured the observed incidence dynamics better than the unfitted model; the hierarchical model was especially preferred for its ability to capture the decline in incidence observed in all regions over time. The unfitted and regionally fitted parameters showed a tendency to increase incidence during the later part of the observation period. Both regional and hierarchical fits were best in the North and Midwest. No set of parameters was able to capture the high peak in PB incidence in the Northeast and Southeast.

All regional estimates of R_e_ had a median value of 1.3, with ranges of 0.97 to 1.7. No region was predicted to eliminate HD as a public health risk within 100 years. Median values of predicted prevalence in the year 2050 ranged from 0.0007 in the South to 0.0045 in the Midwest; this represents a slight increase from the observed prevalence in 2000.

The simulation study, in which fitted values were used to simulate data for each region and the fitting process was repeated, produced almost exactly the same results as the original fitting. Model 3, the simulated model, was preferred for all regions except the Southern region. Parameter values were similar to the input parameters across all regions. The hierarchical model in which all parameters were shared (version 1) was preferred, and the medians (range) of the best-fit parameter distributions were 0.45 (0.24–0.95) for β_M_, 0.27 (0–0.95) for β_P_, 0.13 (0.046–0.29) for γ_M_, and 0.09 (0.03–0.20) for γ_P_. The transmission values were higher than the input parameters, and the transition values were lower, but the distributions were similar.

## Discussion

This study reports the best fit of the previously published mathematical models and parameters for fitting incidence of Hansen’s Disease, in both multibacillary and paucibacillary form, in Brazil. This is the first study to directly compare the different suggested models of HD to field data. By identifying and parameterizing the best-fit model (the model for which the distance between the estimated and observed incidence is smallest), this study provides a guideline for studying control and prevention of HD in an endemically infected country and suggests further improvements to mathematical models of HD.

The ABC algorithm was found to be effective for selecting the appropriate model and marginally adequate for improving the fit for most of the models simulated. This algorithm has been applied to several infectious disease models [17–20], but only one with 2 outcome variables to track [25] (in this case, incidence of PB and MB cases). Use of ABC’s non-likelihood-based approach allowed for easier use of more data, which improved the model fit. However, the algorithm experienced difficulty with the interconnected nature of the parameters; the high negative correlation between transmission and transition parameters lead to a tendency to overestimate one and underestimate the other. This could be corrected if better field estimates of at least one set of parameters were available, to decrease the range of the current, somewhat uninformative, priors. Validation with extreme values resulted in poor fitting, which could be a result of insufficient particles to capture the extremes of the prior distribution. The simulation study, however, found that the algorithm was able to consistently reproduce the simulated values if fitted parameter values were used to simulate the data. This indicates that the algorithm is sufficient to fit realistic parameter values, and is consistent in identifying the best-fit model.

There have been questions with regard to the use of ABC for model selection, especially when applied to insufficient summary statistics [26,27]. Although many of these caveats are related to the joint estimation of models and parameters, as opposed to the separate estimation of parameters for each model applied here, we were aware of the potential for model selection to fail. For this reason, we chose to perform validation tests (see S2 File) with simulated data. This validation found that our summary statistic was able to select the appropriate (simulated) model in most cases. We also considered using a combination of summary statistics, in which the distance between simulated and observed incidence was calculated separately for MB and PB cases and the rejection step required both distance functions to be below their individual thresholds. However, this did not improve model fit or change model selection results during validation trials (data not shown), so the more efficient joint summary statistic was used.

One further difficulty to the fitting of these models was in selecting the initial values for each compartment. As prevalence estimates were available, these were at least able to set the known numbers of PB and MB cases. However, the numbers of latent, dormant, recovered, and undetected cases could not be determined from the available data. This led to the empirical approach applied here (S1 File). The initial values found using this approach were sensitive to the assumed parameter values, which could bias the model fitting process. In order to minimize this, we applied the 2-step approach, with a preliminary model fitting using initial values based on the unfitted parameters. The preliminary fitted parameters were used to determine a new set of initial values, which were then used for the final model fitting. This empirical 2-step approach should decrease the bias of the unfitted parameters while providing reasonable estimates to the true initial values.

In the initial validation test, the algorithm resulted in a preference for the simulated model in all but Model 3, which was the model found to be preferred with the estimation based on observed data. This could be a result of the assumptions necessary in model fitting. In the validation test, all assumptions about model parameters are known to be true, and so will not bias the results of the fitting, allowing more complicated models to reproduce the results of a simple model. In the fitting based on observed data, all unfitted parameters are assumed to be known, but there is a possibility that some may be wrong. As Model 3 has the fewest unfitted parameters, it is the least biased by this uncertainty.

For most regions, except the South, Model 3 was found to have the best fit. The Southern region preferred Model 4 slightly. Model 3 was the simplest, which may explain the preference; there are fewer unfitted parameters to influence the results of the fitting. However, this model does not allow for cure of MB disease, which is known to occur and which all other models allow. It could be that other models underestimate the amount of time that MB individuals are infectious, and that this value (represented by α_M_ in Models 1, 2, and 4 and by φ_M_ in Models 5 and 6) should also be fitted. The current value, however, assumes that the average MB case will become non-infectious in 5 years (in Models 1, 2, and 4) or 2 years (in Model 6), which are reasonably in line with current assumptions; Model 5 assumes a very low case-finding rate, and so is unlikely to be underestimating the length of infectiousness in MB cases. As the amount of time spent infectious would implicitly include the case-finding rate in Models 1–4, and that value would change with differing control programs, it could be appropriate to find better estimates of the recovery rate in future studies. In the present study, the number of observations is insufficient to fit many correlated parameters.

The best-fit parameters, regionally and by hierarchical model, show useful patterns. The transmission parameter for MB cases (β_M_) was almost always higher than that for PB cases (β_P_). This was expected, and reinforces the existing belief that MB cases are more infectious than PB cases, but the difference in the parameter values is smaller than expected. In addition, the transition rate to MB (γ_M_) is slightly higher than the transition rate to PB (γ_P_). As these models are formulated, that indicates that slightly more infections result in MB cases than PB cases, which was observed in the data from Brazil (Fig 2). However, it is known that PB cases can be self-healing and may be under-reported, and so this model (based on reported cases) may be underestimating the true γ_P_, which may also bias estimates of β_M_ and β_P_. It may be useful to configure a model that would take into account the prevalence of genetic susceptibility for MB, allowing for a more accurate representation of the 2-path model that is becoming more accepted for mycobacterial diseases [9].

The hierarchical model in which all parameters were shared was found to provide the best overall fit. The best-fit models were able to dramatically improve the estimated incidence of PB and MB cases in several regions (Figs 5 and 6). In some regions, the regional model showed a better posterior estimation for MB cases; this indicates that there is regional variation in HD dynamics, likely due to regional variations in health and socioeconomic factors [28] as well as regional differences in case detection rates. The North and Northeast regions, for instance, showed much lower transition rates than the other regions, possibly related to lower true case detection rates in these regions. This may also have resulted in the fitting of much higher transmission rates for the Northeast, as cases took longer to become infectious.

The model was overall a poor fit for PB cases the Southeast and Northeast. These regions had later peak incidence, indicating that control programs may have changed during the time period under study. That would cause a poor fit for all parameters, as the transmission rates depend on hygiene, prophylaxis, and vaccination rates and the transition rates depend on case finding and treatment rates.

Based on the results of this study, the 5 regions of Brazil are not progressing towards elimination; the mean and range of the posterior distribution of R_e_ is 1.3 (0.97–1.7), barely touching 1. While an increase in prevalence is predicted, this is possibly a discrepancy between the model structure, which does not allow for recovery from MB cases, and the public health authority, which assumes all cases to be recovered at the end of treatment. The observations being fitted and the criterion for elimination, incidence, would not be affected by this discrepancy. With the current programs remaining unchanged, the model predicts that most regions of Brazil cannot eliminate HD as a public health problem within 100 years. Improved control programs will be needed if the goal of elimination is to be reached.

## Supporting Information

S1 Fig Posterior distributions of 4 parameters fitted to 6 models of Hansen’s Disease using data simulated by each of the 6 models. Labels indicate source of simulation (s) and model fitted (m) as *s*.*m*, with box color representing simulated model. Boxes show median (central line), interquartile range (box) and range (whiskers). The red line indicates the simulated value.
(TIF)

S2 Fig Posterior distributions of 4 parameters fitted to 6 models of Hansen’s Disease using data simulated by the matching model with 16 different combinations of parameters. Labels indicate parameter set (s) and model (m) as *m*.*s*, with box color representing model. Boxes show median (central line), interquartile range (box) and range (whiskers). The red line indicates the simulated value.
(TIF)

S1 File Empirical Determination of Initial Population State. (DOCX)

S2 File Validation of the Approximate Bayesian Computation algorithm for each HD model. (DOCX)

## References

[1] Bennett B, Parker DL, Robson M. Leprosy: steps along the journey of eradication. Public Heal Rep. University of Medicine and Dentistry of New Jersey School of Public Health, Piscataway, NJ, USA. 2008;123: 198–205. 10.1177/003335490812300212 PMC2239329 18457072

[2] Goulart LR, Goulart IMB. Leprosy pathogenetic background: a review and lessons from other mycobacterial diseases. ArchDermatolRes. 2009;301: 123–137. 10.1007/s00403-008-0917-3 19043725

[3] Saunderson PR. Leprosy elimination: not as straightforward as it seemed. Public Heal Rep. American Leprosy Missions, Greenville, SC 29601, USA. 2008;123: 213–216. 10.1177/003335490812300214 PMC2239331 18457074

[4] Leprosy fact sheet (revised in February 2010). Wkly Epidemiol Rec. 2010;85: 46–48. 20151495

[5] Penna MLF, de Oliveira MLW, Penna GO. The epidemiological behaviour of leprosy in Brazil. Lepr Rev. Consultant to the Brazilian National Hansen’s Disease Control Program, Secretariat of Health Surveillance, Federal Ministry of Health, Rio de Janeiro, Brazil. 2009;80: 332–344. Available: http://www.ncbi.nlm.nih.gov/pubmed/19961107 19961107

[6] IDB Brasil. Indicadores e Dados Básicos—Brasil [Internet]. 2012 [cited 7 Feb 2014]. Available: http://tabnet.datasus.gov.br/cgi/idb2012/matriz.htm

[7] Misra R. Leprosy: A Reference Guide For Medical Practitioners, Programme Managers And Leprosy Workers. Concept Publishing Company; 1993.

[8] Lechat M, Misson J, Vellut C, Misson C, Bouckaert A. Un modèle épidémiométrique de la lèpre. Bull World Health Organ. 1974;51: 361–373. Available: http://www.ncbi.nlm.nih.gov/pmc/articles/PMC2366301/ 4549490 PMC2366301

[9] Meima A, Gupte MD, van Oortmarssen GJ, Habbema JDF. SIMLEP: a simulation model for leprosy transmission and control. Int J Lepr other Mycobact Dis. INTERNATIONAL JOURNAL OF LEPROSY; 1999;67: 215–236. Available: http://www-fgg.eur.nl/medbib/EUR-diss/040428_Meima_A/04.pdf 10575401

[10] Mushayabasa S, Bhunu CP, Dhlamini M. Understanding non-compliance with WHO multidrug therapy among leprosy patients: insights from a mathematical model In: Mushayabasa S, Bhunu C, editors. Understanding the Dynamics of Emerging and Re-Emerging Infectious Diseases using mathematical models. Kerala, India: Transworld Research Network; 2012 pp. 1–22. Available: http://www.trnres.com/ebook/uploads/steadycontent/T_1354259023Steady1.pdf

[11] Mushayabasa S, Bhunu CP. Modelling the effects of chemotherapy and relapse on the transmission dynamics of leprosy. Math Sci. 2012;6: 12 10.1186/2251-7456-6-12

[12] Chiyaka ET. Theoretical Assessment of the Transmission Dynamics of Leprosy. Appl Math. 2013;04: 387–401. 10.4236/am.2013.42059

[13] Meima A, Irgens LM, van Oortmarssen GJ, Richardus JH, Habbema JDF. Disappearance of leprosy from Norway: an exploration of critical factors using an epidemiological modelling approach. Int J Epidemiol. 2002;31: 991–1009. 12435774 10.1093/ije/31.5.991

[14] Blok DJ, de Vlas SJ, Fischer EAJ, Richardus JH. Mathematical Modelling of Leprosy and Its Control. Adv Parasitol. 2015;87: 33–51. 10.1016/bs.apar.2014.12.002 25765193

[15] Gillis TP. Is there a role for a vaccine in leprosy control ? Lepr Rev. 2010;78: 338–342. Available: http://www.ncbi.nlm.nih.gov/pubmed/18309707 18309707

[16] Nery JS, Pereira SM, Rasella D, Penna MLF, Aquino R, Rodrigues LC, et al Effect of the brazilian conditional cash transfer and primary health care programs on the new case detection rate of leprosy. PLoS Negl Trop Dis. 2014;8: e3357 10.1371/journal.pntd.0003357 25412418 PMC4239003

[17] Lu Z, Mitchell RM, Smith RL, Karns JS, Van Kessel JS, Wolfgang DR, et al Invasion and transmission of Salmonella Kentucky in an adult dairy herd using approximate Bayesian computation. BMC Vet Res. 2013;9: 245 10.1186/1746-6148-9-245 24304969 PMC4235045

[18] Conlan AJK, McKinley TJ, Karolemeas K, Pollock EB, Goodchild A V, Mitchell AP, et al Estimating the hidden burden of bovine tuberculosis in Great Britain. PLoS Comput Biol. 2012;8: e1002730 10.1371/journal.pcbi.1002730 23093923 PMC3475695

[19] Cook AR, McKinley TJ, Deardon R. Inference in Epidemic Models without Likelihoods. Int J Biostat. 2009;5: 24 10.2202/1557-4679.1171

[20] Toni T, Stumpf MPH. Simulation-based model selection for dynamical systems in systems and population biology. Bioinformatics. 2010;26: 104–110. 10.1093/bioinformatics/btp619 19880371 PMC2796821

[21] Liepe J, Kirk P, Filippi S, Toni T, Barnes CP, Stumpf MPH. A framework for parameter estimation and model selection from experimental data in systems biology using approximate Bayesian computation. Nat Protoc. Nature Publishing Group; 2014;9: 439–56. 10.1038/nprot.2014.025 PMC5081097 24457334

[22] Toni T, Welch PD, Strelkowa N, Ipsen A, Stumpf MPH. Approximate Bayesian computation scheme for parameter inference and model selection in dynamical systems. J R Soc Interface. 2009;6: 187–202. 19205079 10.1098/rsif.2008.0172 PMC2658655

[23] Beaumont MA, Zhang W, Balding DJ. Approximate Bayesian computation in population genetics. Genetics. 2002;162: 2025–2035. Available: http://www.genetics.org/content/162/4/2025.short 12524368 10.1093/genetics/162.4.2025 PMC1462356

[24] WHO. Eliminate Leprosy as a Public Health Problem [Internet]. 1st ed Leprosy Elimination Group, editor. Geneva: World Health Organization; 2000 Available: http://www.who.int/lep/resources/Guide_Int_E.pdf?ua=1

[25] Brooks-Pollock E, Roberts GO, Keeling MJ. A dynamic model of bovine tuberculosis spread and control in Great Britain. Nature. Nature Publishing Group; 2014;511: 228–231. 10.1038/nature13529 25008532

[26] Robert CP, Cornuet J-M, Marin J-M, Pillai NS. Lack of confidence in approximate Bayesian computation model choice. Proc Natl Acad Sci. 2011;108: 15112–15117. 10.1073/pnas.1102900108 21876135 PMC3174657

[27] Barnes CP, Filippi S, Stumpf MPH, Thorne T. Considerate approaches to constructing summary statistics for ABC model selection. Stat Comput. 2012;22: 1181–1197. 10.1007/s11222-012-9335-7

[28] Queiroz JW, Dias GH, Nobre ML, De Sousa Dias MC, Araújo SF, Barbosa JD, et al Geographic information systems and applied spatial statistics are efficient tools to study Hansen’s disease (leprosy) and to determine areas of greater risk of disease. Am J Trop Med Hyg. Health Post-Graduate Program, Department of Internal Medicine, Health Science Center; Department of Biochemistry, Bioscience Center, Universidade Federal do Rio Grande do Norte, Natal, RN, Brazil. jwq@supercabo.com.bn; 2010;82: 306–14. 10.4269/ajtmh.2010.08-0675 PMC2813173 20134009

