# Supplementary figures and images for: Use of Approximate Bayesian Computation to Assess and Fit Models of Mycobacterium leprae to Predict Outcomes of the Brazilian Control Program
Source: PLoS One. 2015 Jun 24;10(6):e0129535. doi: 10.1371/journal.pone.0129535 (PMC4479607; doi:10.1371/journal.pone.0129535)

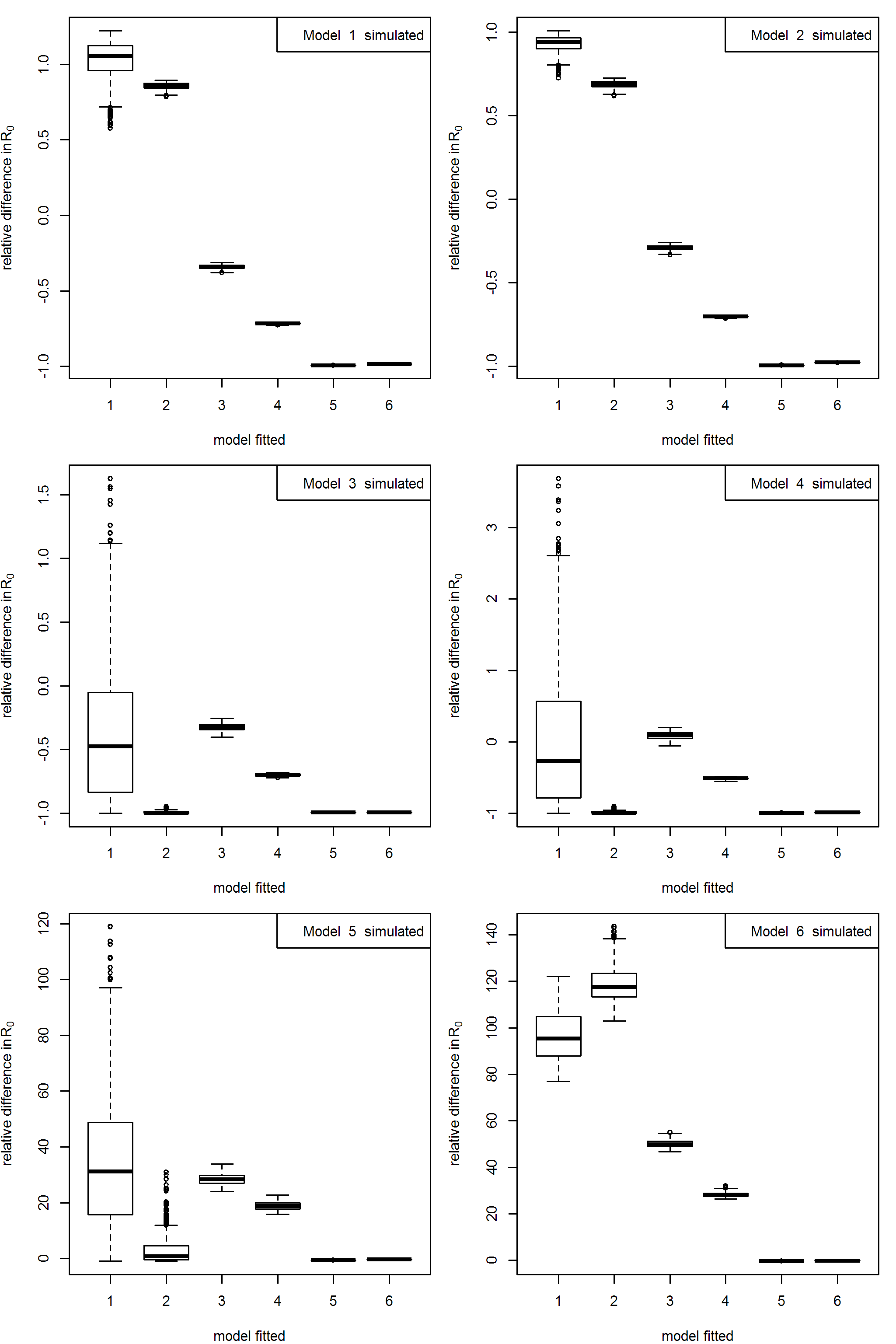

Supplement: S1 Fig — Labels indicate source of simulation (s) and model fitted (m) as s.m, with box color representing simulated model. Boxes show median (central line), interquartile range (box) and range (whiskers). The red line indicates the simulated value. (TIF) [file pone.0129535.s001.tif]

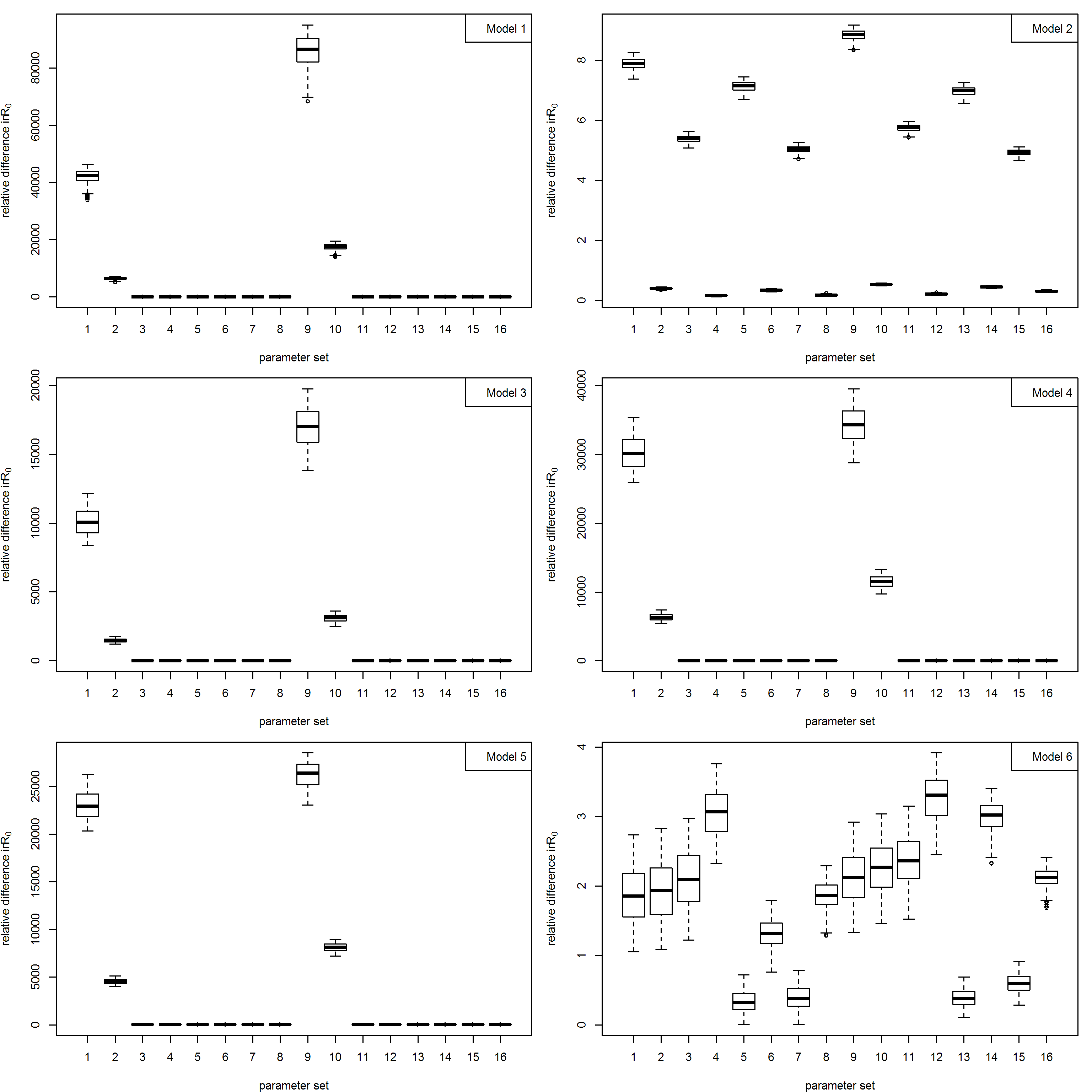

Supplement: S2 Fig — Labels indicate parameter set (s) and model (m) as m.s, with box color representing model. Boxes show median (central line), interquartile range (box) and range (whiskers). The red line indicates the simulated value. (TIF) [file pone.0129535.s002.tif]
