# Supplementary material for: Use of Approximate Bayesian Computation to Assess and Fit Models of Mycobacterium leprae to Predict Outcomes of the Brazilian Control Program
Source: PLoS One. 2015 Jun 24;10(6):e0129535. doi: 10.1371/journal.pone.0129535 (PMC4479607; doi:10.1371/journal.pone.0129535)
Supplement: S1 File — (DOCX) [file pone.0129535.s003.docx]

**S1: Empirical Determination of Initial Population State**

As there were insufficient data to allow simultaneous fitting of the parameters of interest and the initial values in each state of each model (data not shown), the initial population state was determined empirically. This was an iterative process with 2 stages.

Current cases (the product of the prevalence and the population, N*prev) were distributed between MB and PB compartments proportionately to P(MB), the average proportion of new cases being MB across the observation period. The relationship between the number of individuals in the R state and the number of cases (π_R_) was set by running each model to a steady state value and finding the predicted value.

In the first stage, each model was run at the national level using the unfitted (assumed) values for the model and the estimated incidence was compared to the observed incidence. The regional relationships between the number of cases and the number of individuals in the E state (π_e_) were adjusted manually until the estimated incidence in the first year of observation (2000) matched the observed PB incidence for that year within a certain level of tolerance (*tol*),

$$tol_{r}=0.1*inc_{r}^{PB}$$

or the best result with π_e_≥0.001. For Model 2, the number of individuals in the E_D_ state was also determined in this way (π_ed_), and for Model 6, the numbers of MB and PB individuals in their respective treated and recalcitrant categories were also determined in this way (π_mt_, π_pt_).

Using the values from the first stage, the model was fitted regionally to the observed data and posterior estimations were made from running each model with a weighted sampling of its posterior distribution by region. The regional π_e_ were again adjusted manually until the mean estimated PB incidence in the first year of observation (2000) matched the observed incidence for that year within the same tolerance {3}. The resulting values of all π_i_ were then used in the final fitting of the model.
