# Supplementary material for: Use of Approximate Bayesian Computation to Assess and Fit Models of Mycobacterium leprae to Predict Outcomes of the Brazilian Control Program
Source: PLoS One. 2015 Jun 24;10(6):e0129535. doi: 10.1371/journal.pone.0129535 (PMC4479607; doi:10.1371/journal.pone.0129535)
Supplement: S2 File — (DOCX) [file pone.0129535.s004.docx]

**S2: Validation of the Approximate Bayesian Computation algorithm for each HD model**

Each of the models was simulated using the parameters listed in Table 2. The initial population for these simulations was produced using the population size, population demographics, and observed number of cases in treatment in Northeastern Brazil in 2000, creating 6 sets of simulated incidences. These incidence data were used as observational input for the ABC algorithm based on each of the possible models in turn, producing 36 output sets. For each input model, the Bayes Factor (BF) was calculated for the appropriate output model as compared to each of the other models by taking the ratio of the summed distances for each model’s final set of particles. Posterior distributions of all estimated parameters were also determined for each model with each simulated data set. To determine model sensitivity to input values, the same situation was simulated using a range of input values for the estimated parameters, and posterior distributions were determined for each of the 6 models across the data sets simulated by the matched model. For all posterior distributions, R_0_ was calculated for the model fitted, using the calculation of R_0_ provided by the source paper. Model 6 was not tractable to analytical calculation of R_0_, so the numerical solution of the Jacobian matrix was used to calculate R_0_. The relative difference in R_0_ was calculated as $\left(R_{0}^{f}-R_{0}^{s}\right)/R_{0}^{s}$, where *f* refers to the fitted value and *s* refers to the simulated value.

The Bayes Factors from the validation trials for model sensitivity are shown in **Table 1 in S2 File**. In almost every case, the Bayes Factor for the model used to simulate the data was higher than for most other models. The major exception was Model 3, which failed to be preferred to any model other than Model 2. The distributions of the relative difference in R_0_ from the validation trials examining model sensitivity for the ABC algorithm are shown in **S3 Figure**. Models 3 and 4 were better able to reproduce the true value of R_0_ for all but the most complicated model. Models 1 and 2 consistently overestimated the R_0_, especially for data simulated by Models 5 and 6. Models 5 and 6 consistently underestimated the R_0_ for data simulated by Models 1-4. Joint posterior distribution graphs were examined for all parameter pairs to check for multimodal posterior distributions (none noted) and correlation (see below).

The distributions of the relative difference in R_0_ from the validation trials examining parameter sensitivity are shown in **S4 Figure**. Models 1, 3, 4, and 5 all highly overestimated the R_0_ when both β_P_ and γ_M_ were small. Model 2 overestimated the R_0_ when β_M_ was small. Model 6 slightly overestimated the R_0_ in all circumstances, but more so when γ_M_ was small.

The correlation between the transmission and transition parameters was small for Model 1, but for all other models the correlation was considerable, with a median correlation of -0.16 for MB parameters and -0.79 for PB parameters (data not shown). The overall acceptance ratio (number of prior particles included in the posterior) ranged from 0.006 to 0.014, with an acceptance ratio per set of 0.46 to 0.67. This could result in poor coverage of the parameter space; the use of 100,000 iterations per set increased the total number of initial particles included in the posterior to minimize that effect.

The results of the validation shows that the ABC algorithm is able to aid in model selection, but is not able to reproduce the exact simulated parameter values. However, posterior fit can be improved by use of the ABC algorithm.

Table 1: Bayes Factor from validation trials for ABC fitting of 6 proposed HD models. Distance values from the final set were summed for each model fitted, and the Bayes Factor was calculated as the ratio of the summed distance of each model to the summed distance of the model that simulated the data set. Each value is a pairwise comparison of the strength of evidence for the model simulated (row) against the model fitted (column). Values in bold were considered strongly favorable, while values in italics are considered weakly favorable.

| Model | Model Fitted | | | | | |
| --- | --- | --- | --- | --- | --- | --- |
| simulated | 1 | 2 | 3 | 4 | 5 | 6 |
| 1 | *1* | *3.7* | *1.5* | *1.1* | *1.3* | 0.86 |
| 2 | *1.2* | *1* | 0.93 | *1.1* | *1.5* | *1.1* |
| 3 | 0.0025 | *2.8* | *1* | 0.27 | 0.017 | 0.28 |
| 4 | 0.021 | *14* | *2.8* | *1* | 0.093 | *1.3* |
| 5 | *16* | **490** | **100** | **60** | *1* | *11* |
| 6 | *1.1* | **69** | *9.6* | *4.4* | *1.2* | *1* |
